# Model-Based Feature Extraction and Classification for Parkinson Disease Screening Using Gait Analysis: Development and Validation Study

**DOI:** 10.2196/65629

**Published:** 2025-04-08

**Authors:** Ming De Lim, Tee Connie, Michael Kah Ong Goh, Nor ‘Izzati Saedon

**Affiliations:** 1 Faculty of Information Science and Technology Multimedia University Melaka Malaysia; 2 Department of Medicine Faculty of Medicine Universiti Malaya Kuala Lumpur Malaysia

**Keywords:** model-based features, gait analysis, Parkinson disease, computer vision, support vector machine

## Abstract

**Background:**

Parkinson disease (PD) is a progressive neurodegenerative disorder that affects motor coordination, leading to gait abnormalities. Early detection of PD is crucial for effective management and treatment. Traditional diagnostic methods often require invasive procedures or are performed when the disease has significantly progressed. Therefore, there is a need for noninvasive techniques that can identify early motor symptoms, particularly those related to gait.

**Objective:**

The study aimed to develop a noninvasive approach for the early detection of PD by analyzing model-based gait features. The primary focus is on identifying subtle gait abnormalities associated with PD using kinematic characteristics.

**Methods:**

Data were collected through controlled video recordings of participants performing the timed up and go (TUG) assessment, with particular emphasis on the turning phase. The kinematic features analyzed include shoulder distance, step length, stride length, knee and hip angles, leg and arm symmetry, and trunk angles. These features were processed using advanced filtering techniques and analyzed through machine learning methods to distinguish between normal and PD-affected gait patterns.

**Results:**

The analysis of kinematic features during the turning phase of the TUG assessment revealed that individuals with PD exhibited subtle gait abnormalities, such as freezing of gait, reduced step length, and asymmetrical movements. The model-based features proved effective in differentiating between normal and PD-affected gait, demonstrating the potential of this approach in early detection.

**Conclusions:**

This study presents a promising noninvasive method for the early detection of PD by analyzing specific gait features during the turning phase of the TUG assessment. The findings suggest that this approach could serve as a sensitive and accurate tool for diagnosing and monitoring PD, potentially leading to earlier intervention and improved patient outcomes.

## Introduction

### Background

Parkinson disease (PD) is a common neurological disease that affects millions of individuals worldwide. This disorder gradually impairs a person’s ability to move their body, resulting in a variety of crippling symptoms, including tremors, stiff muscles, and sluggish motions. To effectively manage PD and provide timely measures, accurate and early diagnosis is essential.

Traditionally, clinical evaluations conducted by medical professionals, which can be arbitrary and inconsistent, have been used to diagnose PD. In distant or underdeveloped areas, access to specialized PD care may be restricted, delaying diagnosis and therapy. The prevalence of PD is predicted to rise as the world’s population ages, placing additional demand on health care resources and highlighting the need for easily available and effective diagnostic methods.

In this regard, current technology has shown promise in addressing the difficulties associated with PD diagnosis, especially in the areas of machine learning and deep learning. The limits of conventional clinical procedures can be overcome with the promise of early and objective identification provided by machine learning techniques.

This study investigated the use of machine learning for PD identification with a focus on gait characteristics. The goal was to develop a simple and noninvasive method for screening PD symptoms early through analyzing gait patterns.

In this study, kinematic features during the turning phase of the timed up and go (TUG) assessment were extracted and analyzed. Gait variabilities occurring during body turning are more easily identified, as turning involves complex motor coordination and balance adjustments, making it a challenging movement. This increased complexity can accentuate subtle abnormalities in gait patterns that might not be as apparent during straight walking. Turning is particularly difficult for individuals with PD, as it requires precise control and stability, often revealing difficulties such as freezing of gait (FoG), reduced step length, and asymmetrical movements. Therefore, analyzing the turning phase allows for a more sensitive and accurate detection of PD symptoms, providing a reliable indicator of whether a participant exhibits PD-related gait abnormalities. This focus enhances the effectiveness of the diagnostic tool, which contributes to the facilitation of early and accurate identification of PD.

### Conventional Methods

Challa et al [1] proposed an advanced predictive model for PD by using machine learning algorithms, such as multilayer perceptron, boosted logistic regression, random forest, and BayesNet. Their investigation applied the Parkinson Progression Markers Initiative dataset, an extensive dataset containing data from patients with PD and healthy participants. The experimental results showcased remarkable performance improvements over existing methods, boasting accuracy rates of 96.09% for training and 95.45% for testing in the multilayer perceptron algorithm, 96.5854% for training and 96.02% for testing in the BayesNet algorithm, 95.45% for training and 94.87% for testing in the random forest algorithm, and the highest accuracy achieved by the boosted logistic regression algorithm with 97.159% for training and 96.97% for testing. The area under the curve (AUC) of the receiver operating characteristic curve reached an impressive 98.9% for the boosted logistic regression algorithm, emphasizing its robust predictive capabilities. This research represents a significant stride in health care, providing a reliable model for early PD prediction, crucial for timely diagnosis and intervention in addressing this global public health challenge.

In 2019, Polat [2] investigated the recognition of FoG cases in individuals with PD, using a logistic regression classifier trained and tested on a dataset comprising 16 samples. The study meticulously assessed the classifier’s performance using a comprehensive set of 10 performance measures, such as accuracy, miss rate, false discovery rate, false positive rate, false omission rate, sensitivity, specificity, precision, and negative predictive value. Impressively, the logistic regression classifier exhibited a noteworthy accuracy of 81.3% in accurately classifying FoG cases. The research further compared the performance of linear regression with 4 alternative models: linear support vector machine (SVM), quadratic SVM, cubic SVM, and k-nearest neighbors (KNN), revealing that the proposed logistic regression model surpassed its counterparts with the highest accuracy of 81.3% in classifying FoG datasets for individuals with PD. This outcome underscores the superior performance of the logistic regression model in the context of FoG classification.

Vidya and Sasikumar [3] conducted a comprehensive study on the application of multiclass SVM in using gait analysis to identify and grade the severity of PD. The researchers used a publicly accessible dataset containing Vertical Ground Reaction Force Sensors and implemented kinematic analysis to extract spatiotemporal features crucial for the diagnostic process. Their suggested framework included a multiregression strategy to normalize gait time series data and a correlation-based feature selection method. A total of 4 distinct SVM kernel functions such as linear, Gaussian, quadratic, and cubic were rigorously evaluated across 3 different walking tests to gauge their performance. Impressively, the quadratic SVM classifier emerged as the most effective, achieving an outstanding average accuracy of 98.65%. This result surpassed existing state-of-the-art methods, showcasing the robustness and efficacy of the proposed SVM-based approach for PD diagnosis and severity rating.

Moreover, Fang [4] performed a study focusing on predicting PD through the application of machine learning techniques. The research extensively compared the accuracy and recall of 3 distinct algorithms: KNN, random forest, and naive Bayesian. Addressing the inherent limitations of equal weighting in traditional KNN, the study introduced an entropy weight method to enhance KNN’s performance, specifically mitigating equal-weighting issues. In addition, the research delved into a voice-based Unified Parkinson Disease Rating Scale (UPDRS) prediction scheme, leveraging algorithms to effectively predict UPDRS scores from voice data of patients with PD. Notably, the study used the University of California Irvine dataset, showcasing that the refined KNN algorithm surpassed its traditional counterpart, achieving a notable accuracy rate increase from 91.8% to 93.8%. This research contributes significantly to the advancement of PD prediction methodologies, particularly emphasizing the pivotal role of improved weighting mechanisms in enhancing algorithmic accuracy.

In addition, Gundala et al [5] conducted a comprehensive study using the random forest algorithm for the recognition of PD using the spiral handwritten dataset. The machine learning technique, widely recognized for its efficacy in processing handwritten designs, was applied by partitioning the dataset into subgroups on the basis of features and constructing decision trees for each feature. The dataset, sourced from the Kaggle website, provided the foundation for training the random forest model. Notably, the algorithm’s strength lies in its ability to combine outputs from multiple decision trees through majority voting, resulting in a remarkable accuracy rate of 91%. The study emphasizes the robustness of the random forest algorithm in accurately identifying PD on the basis of the features extracted from handwritten drawings. The results underscore the algorithm’s effectiveness in leveraging the diversity of decision trees for enhanced predictive accuracy in the context of PD diagnosis.

Moreover, Govindu and Palwe [6] analyzed Multidimensional Voice Program audio data collected from both patients with PD and healthy individuals. Among the machine learning models evaluated, the random forest classifier emerged as the most effective, achieving a detection accuracy of 91.83% and a sensitivity of 0.95. This model outperformed other techniques, such as SVM, KNN and logistic regression, which were also assessed for their classification capabilities. The superior performance of the random forest classifier highlights its robustness and reliability for detecting PD in its early stages, demonstrating the promise of machine learning in enhancing diagnostic accuracy.

### Deep Learning Methods

Pereira et al [7] proposed an innovative method for early identification of PD using a convolutional neural network (CNN) trained on handwritten dynamics data obtained by a smart pen. The CNN effectively learned relevant features from the signals generated during individual exams, enabling discrimination between individuals with and without PD on the basis of these learned features. Experimental results showcased the superiority of the CNN over raw data, with the ImageNet architecture, using 128 times 128 images and a 75% training dataset split, yielding the best overall accuracy of 83.77%. Despite these promising results, the study acknowledged challenges in achieving consistent recognition rates over control individuals, emphasizing the need for further refinement in the proposed approach.

Later, Grover et al [8] introduced a deep learning methodology using a deep neural network (DNN) constructed with TensorFlow and Keras, containing 3 hidden layers with 10, 20, and 10 neurons each and an input layer with 16 units, concluding with an output layer representing the classes *severe* and *nonsevere*. The DNN was trained on the Parkinson telemonitoring voice dataset from the University of California Irvine machine learning repository, comprising biomedical voice measurements from 42 participants. The experiments focused on predicting the severity of PD based on total UPDRS and motor UPDRS scores. The DNN exhibited substantial accuracy improvements over previous research, achieving a classification accuracy of 94.4422% for total UPDRS and 83.367% for motor UPDRS on the training dataset. While test dataset results were comparatively lower, with 62.7335% accuracy for total UPDRS and 81.6667% for motor UPDRS, the proposed DNN classifier showcased enhanced performance compared to previous studies, emphasizing its potential for accurate severity prediction in PD.

Aal et al [9] conducted an optimized approach for early PD detection, using speech features extracted from 2 datasets, dataset 1 and dataset 2, containing recordings from both healthy individuals and patients with PD. Using Mel-frequency cepstral coefficients and delta Mel-frequency cepstral coefficients, the authors proposed a deep learning model combining a recurrent neural network (RNN) with a long short-term memory (LSTM) layer. Comparative evaluation of alternative ML techniques, including SVM, KNN and RNN with stochastic gradient descent, revealed superior performance of the proposed RNN-LSTM model, optimized with the adaptive moment estimation optimizer. The model exhibited remarkable testing accuracy rates of 95.8% on dataset 1 and 90.24% on DS2, accompanied by high recall, precision, and *F_1_*-score on both datasets. Aal et al [9] further demonstrated the model’s superiority over existing methods for PD detection, solidifying its potential as an effective tool for early diagnosis.

Apart from that, Ouhmida et al [10] delved into the early recognition of voice-based PD through the application of CNN and artificial neural network (ANN). Using 2 distinct datasets, the study showcased the superior accuracy of CNN over ANN in their experiments. Dataset 1 encompassed 195 voice recordings from 31 individuals, while dataset 2 featured 240 recordings from 80 participants, with a balanced distribution of 40 patients with PD and 40 healthy individuals. The deep learning models, specifically CNN and ANN, underwent training and testing on both datasets, revealing CNN’s accuracy rates of 93.10% and 88.89% for datasets 1 and 2, respectively. In contrast, the ANN model achieved slightly lower accuracies, with 82.76% for dataset 1 and 72.22% for dataset 2. Ouhmida et al [10] also highlighted the intricate layers comprising each model, with the ANN model featuring 2 hidden layers and the CNN model incorporating convolution, normalization, activation, softmax, and classification layers. The paper expressed an intent to extend the research by exploring additional deep learning methods and implementing a hybrid system integrating diverse techniques and datasets.

Biswas et al [11] proposed an approach for the early recognition of PD by proposing 2 distinct deep learning models tailored for hand-drawn graphics. The first model, a 2D CNN, processed preprocessed images of spirals, circles, and meanders as input, achieving notable accuracies of 83.6% on circles, 61.5% on spirals, and 67.8% on meanders. Another model, an innovative LSTM model, operated on timeline-series signals and demonstrated an overall accuracy of 0.78. Leveraging the NewHandPD dataset for training and testing, the authors conducted 4 comprehensive experiments, presenting the results. The study posited that early PD detection through these advanced models could potentially enhance treatment outcomes and elevate the overall quality of life for affected individuals.

Apart from that, Khaskhoussy and Ayed [12] evaluate the effectiveness of SVM CNN for classifying data obtained from speech tasks. Total 2 types of input data were analyzed: the raw speech signal values and i-vector features of dimensions 100, 200, and 300. The classification performance was assessed using 5 evaluation metrics: accuracy, precision, recall or sensitivity, specificity, and *F*_1_-score. For a test dataset of 28 participants, the approach achieved outstanding results, including 100% accuracy, a precision of 0.99, a recall of 0.98, a specificity of 0.96, and an *F*_1_-score of 0.98. A table summarizing the state-of-the-art methods is presented in Table 1.

**Table 1:** A summary of the state-of-the-art methods.

Study	Methods	Input type	Dataset	Performance
Challa et al [1]	MLP^a^, BayesNet, RF^b^, and boosted logistic regression	Nonmotor symptoms	PPMI^c^ dataset	Best overall accuracy: 96.97%
Polat [2]	Logistic regression	FoG^d^	FoG dataset (Parkinson disease)	81.3%
Vidya and Sasikumar [3]	SVM^e^	Gait features	Gait analysis dataset	98.65%
Fang [4]	KNN^f^	Voice records	UCI^g^ dataset	93.8%
Gundala et al [5]	RF	Handwritten drawings	Kaggle handwritten drawings dataset	91%
Pereira et al [7]	CNN^h^	Handwritten dynamics	Public dataset of handwritten dynamics extracted by a smart pen	Best overall accuracy: 83.77%
Grover et al [8]	DNN^i^	Voice dataset	Parkinson telemonitoring voice dataset	Total UPDRS^j^: train 94.44%, test 62.73% and motor UPDRS: train 83.37%, test 81.67%
Aal et al [9]	RNN^k^-LSTM^l^	Speech features	Dataset 1 and dataset 1^2^	Dataset 1: 95.8% and dataset 12: 90.24%
Ouhmida et al [10]	CNN ANN^m^	Voice	Dataset 1, dataset 2	Dataset 1: 93.10% and dataset 2: 88.89%
Biswas et al [11]	LSTM	Hand drawings	NewHandPD	78.7%

^a^MLP: multilayer perceptron.

^b^RF: random forest.

^c^PPMI: Parkinson Progression Markers Initiative.

^d^FoG: freezing of gait.

^e^SVM: support vector machine.

^f^KNN: k-nearest neighbors.

^g^UCI: University of California Irvine

^h^CNN: convolutional neural network.

^i^DNN: deep neural network.

^j^UPDRS: Unified Parkinson Disease Rating Scale.

^k^RNN-: recurrent neural network.

^l^LSTM: long short-term memory.

^m^ANN: artificial neural network.

## Methods

### Overview

The study used a methodical approach that started with data collection from young adults, older adults, and patients with PD, followed by obtaining consent, particularly from those diagnosed with the disease. The TUG assessment was a key part of the data collection process. Video enhancements and preprocessing were performed to enhance the quality of the videos. After that, the key points on the human body were obtained using a human pose estimation technique. Next, features such as shoulder distance, step and stride lengths, cadence, and speed were extracted to analyze the gait patterns. The Butterworth filter was applied to refine the data, and peaks were identified to calculate steps and turning durations. Finally, SVM [2] was used to distinguish between the different groups based on the extracted features, aiming to improve the prediction and analysis of PD symptoms.

### Dataset

The self-collected dataset comprised video recordings of 28 individuals performing the TUG assessment, a standard test used to evaluate mobility and balance. The dataset comprised 3 distinct cohorts: young adults aged between 21 and 33 years, older individuals aged >60 years, and older individuals diagnosed with PD. Consents were obtained from the participants before data collection. The videos captured various gait patterns, providing a comprehensive set of data for analyzing kinematic features, such as step length, stride length, and joint angles. This dataset allowed for controlled conditions and detailed annotations, ensuring high-quality data for feature extraction and analysis.

### TUG Assessment

**Figure 1:**
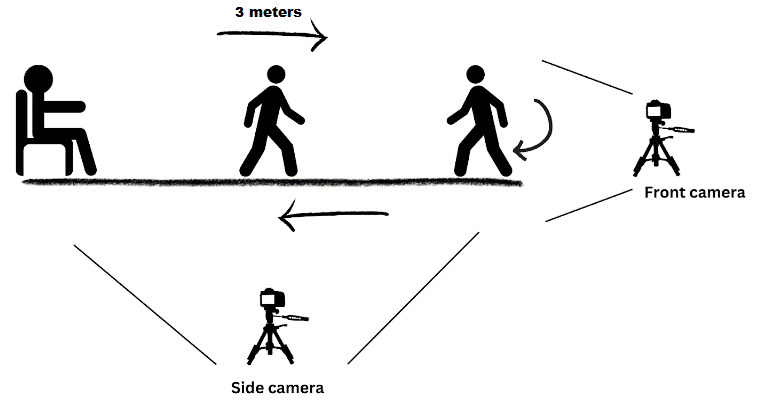
Timed up and go (TUG) assessment shooting site configuration plan.

Figure 1 shows a TUG workflow, outlining the sequential steps involved in the activity. The process began with the individual seated at the designated starting point. Upon initiating the recording, the participant stood up and proceeded to walk a distance of 3 meters in a typical manner. Upon reaching the 3-meter mark, the individual executed a turn, walking back to the starting point, where they concluded the TUG process by returning to a seated position. This comprehensive description encapsulated the entire TUG procedure, providing a clear understanding of the task’s progression. To enhance data capture, 2 phones were used, one on the front and the other on the side.

### Video Enhancement

**Figure 2:**
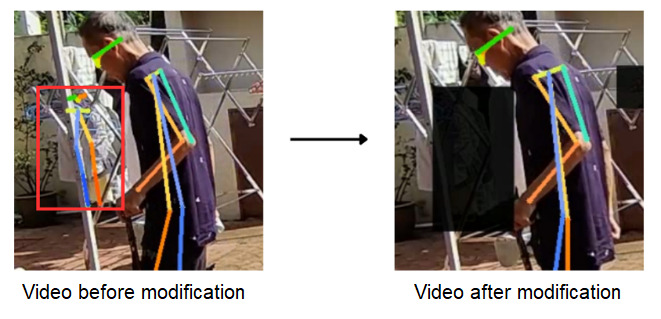
Video enhancement.

The presence of background clutter in the original video introduces noise, which significantly affects the accuracy of letter pose extraction. Applying human pose estimation technique directly to unprocessed video may result in inaccurate keypoint coordinates, affecting the reliability of analysis results. To alleviate this problem, a preprocessing step was crucial. Specifically, the video was subjected to a customized processing method in which a black block was used to mask certain areas susceptible to noise. This strategic approach ensured that no noise interferes with the subsequent process, resulting in more precise and reliable coordination extraction. Figure 2 shows an example of enhancement performed on the video images.

### Human Pose Estimation

**Figure 3:**
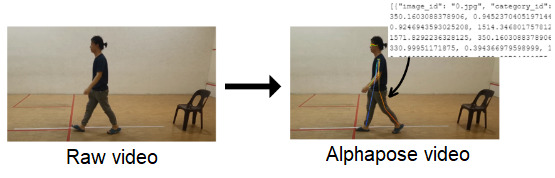
Gait analysis using AlphaPose.

In this study, AlphaPose was used to identify 17 key points from the human body, each corresponding to a specific anatomical location. The process yielded a JSON file containing coefficients for 17 key points. It is important to note that the JSON file, at this stage, has not undergone data processing. The raw file was populated with various elements, including image_id, category_id, key points, and scores within each set. The image_id represented the frame of the video, while the category_id served to identify the object (set as 1 for a person). The key points section contains coordinates for body part locations and corresponding detection confidence, formatted as x1, y1, c1, x2, y2, c2, and so forth, with “c” denoting the confidence score (Figure 3).

### Data Extraction

In the data preprocessing phase, the first step involved loading the JSON file and carefully filtering key points and image IDs. In addition, confidence scores were eliminated. The next focus was to mark the 17 identified body key points, including nose, left eye (LEye), right eye (REye), left ear (LEar), right ear (REar), left shoulder (LShoulder), right shoulder (RShoulder), left elbow (LElbow), right elbow (RElbow), left wrist (LWrist), right wrist (RWrist), left hip (LHip), right hip (RHip), left knee (LKnee), right knee (Rknee), left ankle (LAnkle), and right ankle (RAnkle). Next, according to the specific video file name, key point coordinates for each image ID (frame) were marked, ranging from Nose_x and Nose_y to RAnkle_x and RAnkle_y.

The results from the data preprocessing stage boasted a comprehensive structure, encompassing 36 columns that capture key information. Each column was curated to provide a detailed representation of the dataset. Starting with the file_name, which specified the associated video file, and the image_id indicating the frame or image ID corresponding to each set of key points, the subsequent 34 columns focus on the x and y coordinates of 17 distinct body key points.

### Frame Segmentation

**Figure 4:**
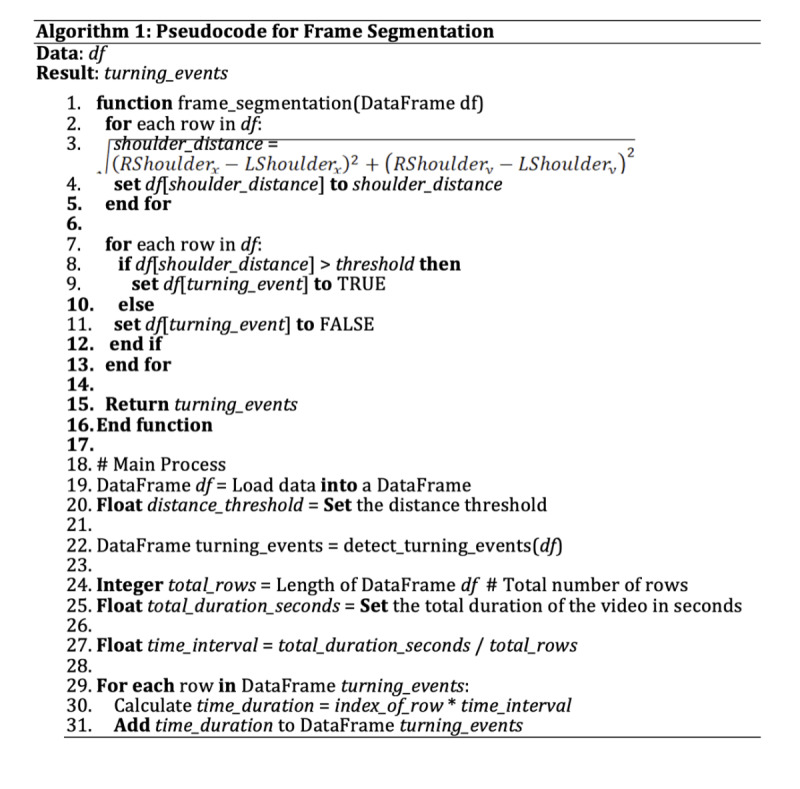
Pseudocode describing the step-by-step procedure for frame segmentation.

Frame segmentation is a process to assign a time interval to each frame in the video which helps to determine the occurrence of a turning event. The total duration of the video is divided by the total number of frames (rows). This calculation yields the time interval per frame, assuming a constant frame rate throughout the video. With this interval calculated, a new column named “time_duration” is added to the DataFrame. Each row in the “time_duration” column is populated with the cumulative time, calculated as the frame’s index in the DataFrame multiplied by the time interval. This method provided a time stamp for each frame, which is essential for synchronizing the data with the video and analyzing the timing of the detected body turning events. The pseudocode presented in Figure 4 describes the step-by-step procedure for frame segmentation.

### Noise Filtering

The Butterworth filter was used as the noise filtering tool in this study. The Butterworth filter is a type of signal processing filter that plays a pivotal role in enhancing the clarity of movement coordinate data extracted from videos. By applying the Butterworth filter to the raw coordinates, high-frequency noise is effectively attenuated, resulting in a smoother trajectory on the graph. This smoothed representation provides a clearer visualization of the participant’s movements, reducing interference from irrelevant fluctuations.

Combined with the Butterworth filter, a peak detection algorithm was used to identify important points on the graph. These peaks correspond to key events in motion, such as steps taken by the participant. The peak identification process helped in extracting basic features such as the number of steps throughout the video. This kind of feature extraction is particularly valuable for specific PD aspects of analysis and quantification. The Butterworth filter is expressed using equation 1:



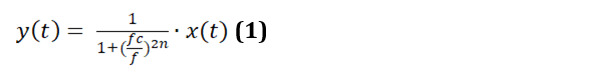



where *y*(*t*) is the filtered signal; *x*(*t*) is the input signal; *fc* is the cutoff frequency; *f* is the frequency of the signal; and *n* is the order of the filter.

In this study, an order of 4 was used for the Butterworth filters because it provides a smooth yet sufficiently steep roll-off in the transition band. While higher-order filters provide sharper roll-offs, they also require more computational resources. Therefore, a fourth-order filter appeared to be a reasonable compromise, being computationally efficient while providing adequate filtering. On the other hand, the peak detection equation is given by equation 2:



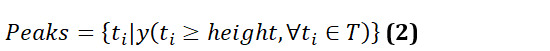



where *y*(*t*) is the filtered signal; *T* is the set of all time points in the signal *y(t)*; *t_i_* represents the time point where a peak occurs; and *height* is the threshold height for peak detection.

**Figure 5:**
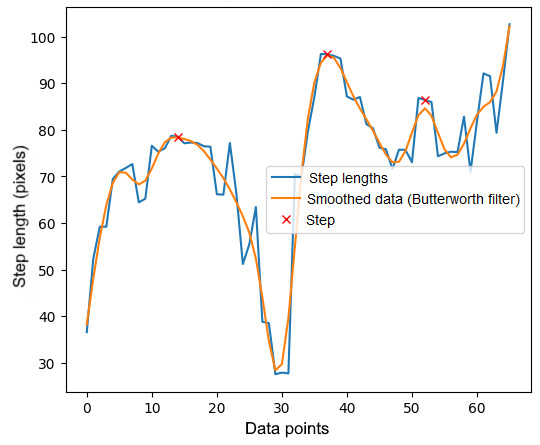
An example of a graph processed with a Butterworth filter, showing peaks.

Only peaks that have an amplitude greater than or equal to the threshold, *h*, are detected and included in the output. In this study, the value for the height parameter was set to 100. An example of the graph for noise filtering with peaks is illustrated in Figure 5. This example demonstrates that the application of the Butterworth filter smooths the signal and yields an accurate step count during body turning.

### PD Recognition Using Model-Based Features

Certain gait features can help identify abnormalities or changes in walking patterns, which are particularly useful for diagnosing and monitoring conditions such as PD. By focusing on the body turning period, we aimed to capture the most challenging part of the gait cycle, where gait variabilities for PD are more easily observed. Turning involves complex motor coordination and balance adjustments, making it a difficult movement. This complexity can accentuate subtle abnormalities in gait patterns that might not be as apparent during straight walking. Therefore, analyzing gait features during turns provides a more sensitive and accurate assessment of PD-related gait abnormalities, enhancing the effectiveness of early diagnosis and monitoring. The gait features used in this study are summarized in Table 2.

**Table 2:** Gait analysis feature definitions and formulas.

Feature	Definition	Formula
Shoulder distance	Average horizontal distance between the left and right shoulders	mean(Shoulder_Distance(LShoulder_x_,LShoulder_y_,RShoulder_x_,LShoulder_y_))
Step length	Average distance between the left ankle and right ankle	mean(Step_Length(LAnkle_x_,LAnkle_y_,RAnkle_x_,RAnkle_y_))
Stride length	Average distance covered in 1 full stride, which consists of 2 steps (1 by each foot)	mean(Stride_Length(RAnkle_x_,RAnkle_y_))
Angle of both knees	Degrees at the knee joint by considering the vectors formed by the knee to hip and knee to ankle points	Θ_Knee_(Hip_x_,Hip_y_,Knee_x_,Knee_y_,Ankle_x_,Ankle_y_)
Angle of both hips	Degrees at the hip joint by considering the vectors formed by the hip to knee and hip to shoulder midpoint	Θ_Hip_ (Shoulder_midₓ_,Shoulder_midy_, Hip_x_. Hip_y_, Knee_x_, Knee_y_)
Symmetrical leg	Degrees for each ankle by considering the vectors formed by the knee to hip midpoint and knee midpoint to hip midpoint	Θ_Leg_(Knee_midₓ_,Knee_midy_, Hip_midₓ_. Hip_midy_, Knee_x_, Knee_y_)
Symmetrical arm	Degrees for each arm by considering the vectors formed by the arm to shoulder midpoint and arm midpoint to shoulder midpoint	Θ_Arm_(Shoulder_midₓ_,Shoulder_midy_, Elbow_midₓ_. Elbow_midy_)
Trunk Angle 1 (vertical)	Degrees by considering the vertical reference vector and the vector from the hip midpoint to the nose	Θ_Trunk1_(Hip_midₓ_,Hip_midy_, Nose_x_. Nose_y_)
Trunk angle 2 (horizontal)	Degrees by considering the vector from the hip midpoint to the shoulder midpoint and a horizontal reference vector	Θ_Trunk2_(Shoulder_midₓ_,Shoulder_midy_, Hip_midₓ_,Hip_midy_)
Shank angle	Degrees at the hip joint by considering the vectors formed by the left ankle to knee midpoint and right ankle to knee midpoint	Θ_Shank_(Ankle_x_,Ankle_y,_Shoulder_midₓ_,Shoulder_midy_)

### Body Turning Duration Calculation

**Figure 6:**
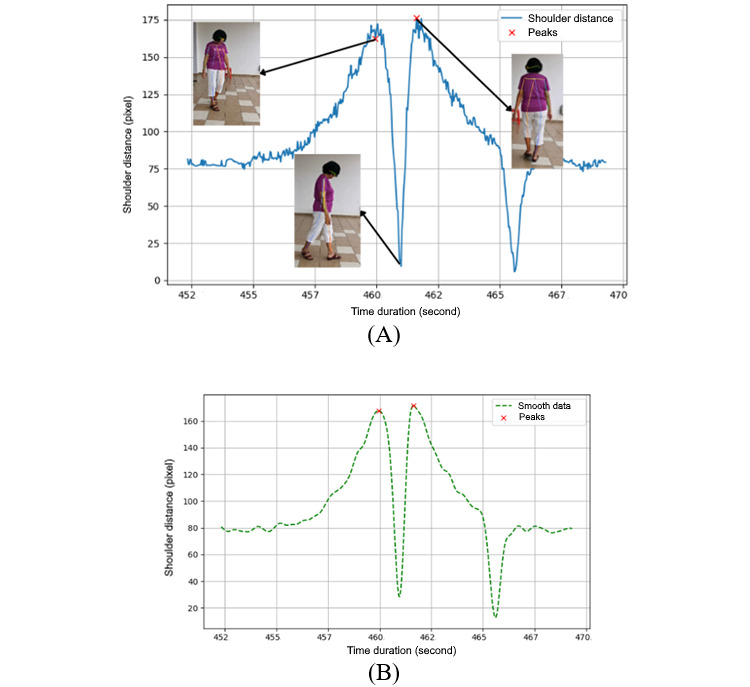
Shoulder distance analysis with peak detection: (A) graph before smoothing and (B) graph after smoothing.

Figure 6 presents 2 graphs related to the analysis of shoulder distance during a body turn. The left graph (Figure 6A) displays the original, unprocessed data, illustrating the raw measurements of shoulder distance over time. The fluctuations observed may correspond to the natural movement variations during a turn. In contrast, the right graph (Figure 6B) exhibits data that have been smoothed using a Butterworth filter, a technique that mitigates short-term fluctuations and reveals the underlying pattern of movement more clearly.

The smoothed data enables effective determination of the peaks of the signal, which identifies the significant turning points. These peaks are highlighted by the red “X” marks in the figure and represent moments where the shoulder distance reaches its maximum, indicating a complete turn or a change in direction. By focusing on the 2 highest peaks, the graph underscores the most substantial turning events, thereby minimizing the potential for misinterpreting minor variations as significant movements.

In the context of shoulder distance, *LShoulder* and *RShoulder* would correspond to the left and right shoulders, with their respective *x* and *y* coordinates. The shoulder distance, *Shoulder_Distance*, which is a measure of how far apart the shoulders are, is calculated as follows (equation 3):



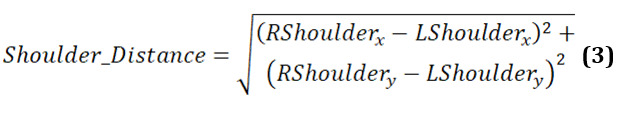



The indexes between these 2 peaks are then taken to determine the exact time the turn occurred. The difference between these peak times is calculated to find the duration between consecutive turning points. Let *T_peak,i_* be the time of the *i*th peak and *n* is the total number of peaks detected. Equation 4 calculates the total time duration between the first and the last peak by adding up the durations between each pair of consecutive peaks,



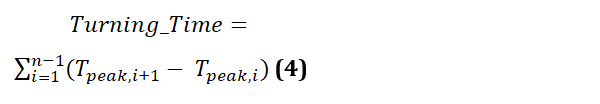



### Ethical Considerations

Ethical approval was granted by the Research Ethics Committee Multimedia University (approval number EA0422022). Informed consent was obtained from all the participants. A statement about this is given in the Methods section. Identifiable features of research participants are not visible in the manuscript. No compensation was provided to the participants.

## Results

### Overview

This section presents the experiment results and discussion for the gait features extracted from the self-collected dataset, focusing on the effectiveness of these features in distinguishing between normal and PD-affected gait patterns using a SVM classifier.

For the experiments, principal component analysis was applied to reduce the dimension of the dataset. The number of components for principal component analysis was set to 8, corresponding to the number of participants with PD. This step helps in capturing the most significant features while reducing computational complexity.

The dataset was additionally divided into a 70:30 ratio. This indicates that the model was trained using 70% of the data, with the remaining 30% set aside for testing and performance evaluation. By splitting the data this way, a significant portion of the data were used to train the model while maintaining enough for a thorough assessment. In total, 10 trials of the experiments were performed, and the average results were recorded.

### Participant Details

There were 3 groups of participants in the self-collected dataset, namely, patients with PD, older adults, and adolescents. A significant portion of the participants (8/28, 29%) were diagnosed with PD, while the remaining participants were healthy (20/28, 71%). Most of the participants were Chinese (24/28, 85%), with a smaller representation of Indian (3/28, 11%) and Malay (1/28, 4%) individuals. There was a higher proportion of male participants (21/28, 75%) compared to female participants (7/28, 25%). Finally, most participants fell within the age range of 60 to 69 years (14/28, 50%), followed by the age range of 20 to 29 years (9/28, 32%), and smaller proportions in other age ranges. These results collectively illustrate the diversity and focus of the study population.

### Total Steps During Body Turning

**Figure 7:**
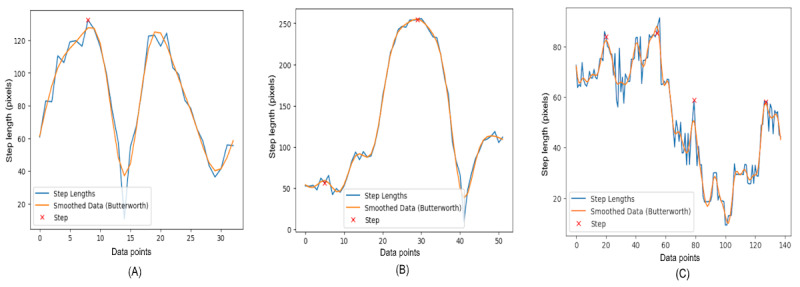
A comparative step analysis by step length between different groups: (A) younger individuals, (B) older individuals, and (C) individuals with Parkinson disease.

Figure 7 presents 3-line graphs that correspond to the step lengths for different demographic groups: younger individuals, older individuals, and individuals with PD. The step lengths are measured during a turning sequence.

The graph of younger individuals (Figure 7A) demonstrates a more variable stepping pattern with pronounced peaks, suggesting agility and a higher range of motion (ROM) during turns. The steps are uneven in length, but the peaks return to baseline quickly, indicating swift changes in direction and a potentially more dynamic gait.

In contrast, the graph of older individuals (Figure 7B) has fewer, more rounded peaks, indicative of a more cautious or steady turning strategy. The step lengths are generally more uniform, with smoother transitions between steps. This could reflect a more deliberate and potentially less stable gait, as is often seen with aging.

The graph of individuals with PD (Figure 7C) demonstrates a significantly different pattern, with smaller step lengths and a higher frequency of steps, which may point to the short, shuffling steps often associated with PD. The peaks are less pronounced and more erratic, highlighting the challenges individuals with PD may face in maintaining a regular stepping pattern.

### Step Length

Step length refers to the distance between the left and right ankle for each step. It is determined by the coordinates of the left ankle (*LAnkle_x_, LAnkle_y_*) and the right ankle (*RAnkle_x_, RAnkle_y_*) at a particular instance in time. The formula to calculate step length is provided in equation 5. This formula gives the straight-line distance between 2 points in a 2D space, such as a video frame or a motion capture system’s coordinate plane.



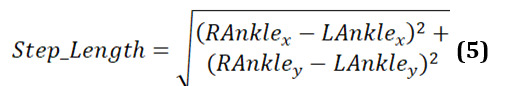



**Figure 8:**
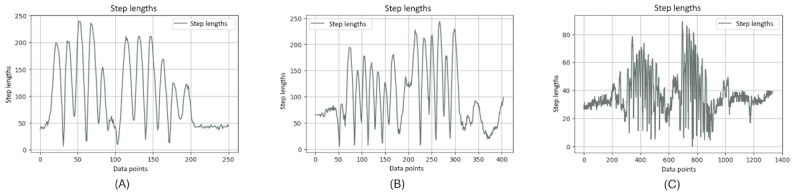
A comparative step length analysis between different groups: (A) younger individuals, (B) older individuals, and (C) individuals with Parkinson disease.

In Figure 8, the graphs illustrate the differences in gait patterns across 3 demographic groups during a TUG test. Each graph plots the variation in step lengths, which reflect the distance between the left and right ankle during individual steps.

The graph of younger individuals (Figure 8A) is characterized by high peaks and deep valleys, indicating significant variability in step lengths. This could suggest a dynamic and robust gait, with the ability to take both long and short steps, perhaps adjusting speed or direction more frequently.

In contrast, the graph of older individuals (Figure 8B) shows a moderate level of variability with somewhat rounded peaks. The reduced height of the peaks compared to the young group suggests shorter steps on average, which may be a sign of a more cautious approach to movement, possibly due to decreased mobility or balance concerns that come with age.

The graph of individuals with PD (Figure 8C) differs markedly from the other 2. It has a much higher frequency of smaller fluctuations, and the overall range of step lengths is noticeably lower. This pattern indicates the short, shuffling steps that are often observed in individuals with PD, reflecting the challenges they face with gait initiation and continuation.

### Stride Length

Stride length is typically defined as the distance covered in one full stride, consisting of 2 steps (one by each foot). The equation to calculate the stride length for each frame is presented in equation 6. It calculates the stride length as the Euclidean distance from a point (possibly the origin) to the coordinates of the right ankle for each frame.



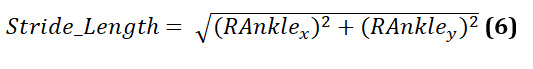



**Figure 9:**
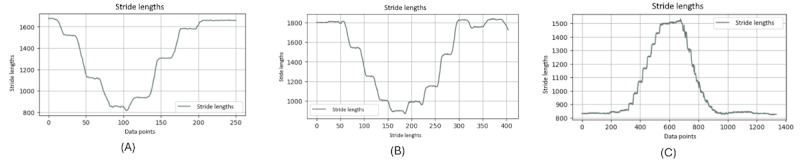
A comparative stride length analysis between different groups: (A) younger individuals, (B) older individuals, and (C) individuals with Parkinson disease.

Figure 9 shows the stride length of the right ankle throughout a single TUG video. The graph exhibits the dynamic nature of stride lengths, with the vertical axis representing the stride length and the horizontal axis corresponding to data points that likely represent sequential frames of the video.

It is noteworthy that this graph clearly distinguishes between 2 types of motion: the rising and falling edges indicate the leg in motion, not in contact with the ground, and the flat regions signify the stance phase where the leg is stationary on the floor, awaiting the next step. The consistency in the flat regions indicates moments when the leg is at rest between strides.

It is evident that the graphs of younger and older individuals (Figures 9A and 9B) display similar frequencies in stride patterns, whereas the graph of the individuals with PD (Figure 9C) significantly diverges. It illustrates a strikingly different frequency in stride lengths, standing out from the more consistent rhythmic patterns observed in the younger- and older-individual groups.

Moreover, from these charts, it is obvious that when younger and older people complete a stride, their feet leave the ground to a greater extent, because a normal stride will take a relatively large range. On the contrary, when patients with PD complete a stride, their feet leave the ground to a relatively smaller extent. The amplitude of the ground is relatively short, which can be considered a symptom of PD.

### Angle of Both Knees

**Figure 10:**
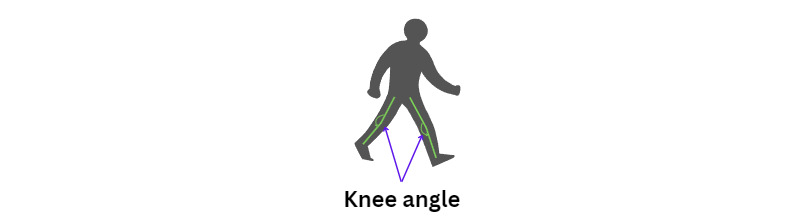
An illustration of the knee angle position.

Analyzing the positions of the hip, knee, and ankle for each leg to determine the angles at both knees can assist in recognizing PD (Figure 10).

The knee angles provide crucial insights into the degree of bending or extension at the knee joints, which are essential for understanding gait patterns. PD often affects movement and posture, leading to distinctive gait abnormalities. By quantifying these knee angles, the calculation helps in detecting and assessing these gait characteristics, aiding in the identification and monitoring of PD symptoms.

Given the knee, hip, and ankle joint positions, the knee angle and the Θ_knee_ can be computed as follows (equation 7):



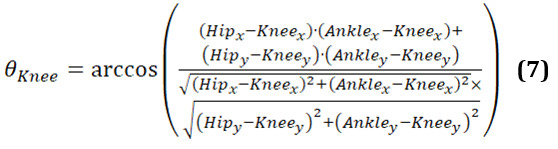



**Figure 11:**
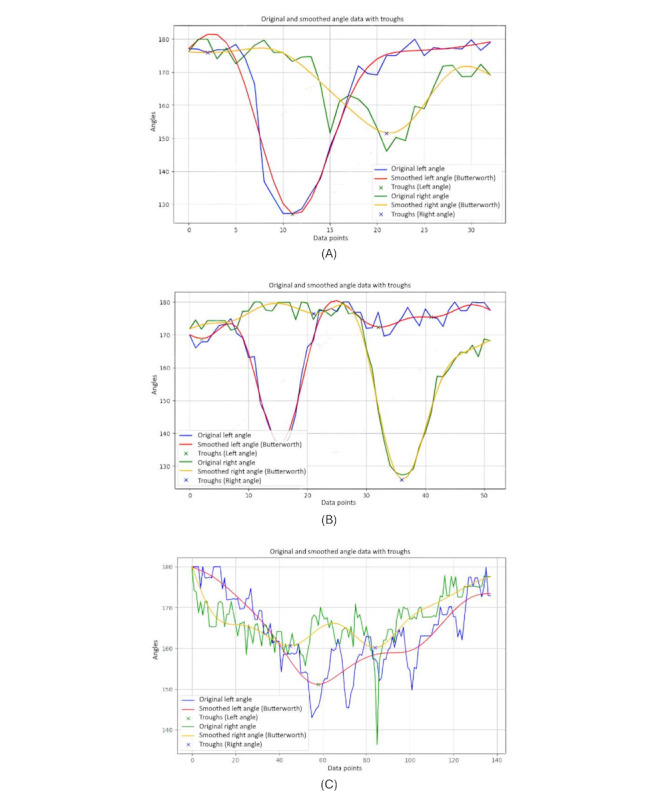
A comparative analysis of the knee angle between different groups: (A) younger individuals, (B) older individuals, and (C) individuals with Parkinson disease.

Figure 11 presents 3-line graphs comparing the knee joint angles of the 3 groups: younger individuals, older individuals, and individuals with PD. The graph of the younger individuals group (Figure 11A) illustrates smooth and regular oscillations in knee joint angles for both the left and right legs. As they turn their body, the angles show significant flexion and extension, reflecting the youthful ability to execute the turn in a single, fluid step. The peaks and troughs are well-defined, indicating robust and agile movements typical of healthy, young individuals. This pattern highlights their efficient and coordinated gait.

In the older-individual group graph (Figure 11B), the knee joint angles are more subdued, with 2 distinct curves for each turn. This indicates that older individuals take 2 steps to complete the body turn, reflecting a more cautious and segmented approach. The movement patterns are slower and less dynamic compared to the younger individual group, with a reduced ROM.

The graph for the group of individuals with PD (Figure 11C) shows highly irregular and erratic knee joint angles. The curves exhibit frequent sharp peaks and drops, indicating inconsistent and disrupted movement as individuals with PD attempt to turn their bodies. These fluctuations reflect the challenges faced by patients with PD, including tremors, rigidity, and difficulty maintaining smooth and coordinated movements. The graph captures their struggle to control knee flexion and extension, leading to a more disordered and interrupted gait pattern during the turning motion.

These graphs highlight clear differences in turning movements: young individuals exhibit fluid, single step turns; older individuals use a careful 2-step approach; and patients with PD show irregular, disrupted turning patterns.

### Angle of Both Hips

**Figure 12:**
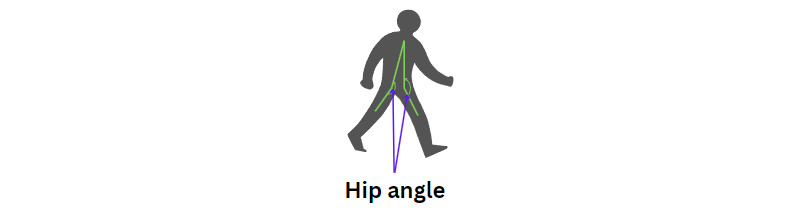
An illustration of the hip angle position.

The ROM angle while turning the body is used to determine the angle of both hips (Figure 12). The positions of the hip, knee, and shoulder are used to find the angle formed at the hip joint. This measurement is crucial for assessing how the hip moves during turns, providing insights into the flexibility and coordination of the lower body. Understanding the hip ROM is especially valuable for identifying movement patterns in different populations, such as detecting mobility issues or assessing gait in individuals with PD.

The hip angle is calculated as follows (equation 8):



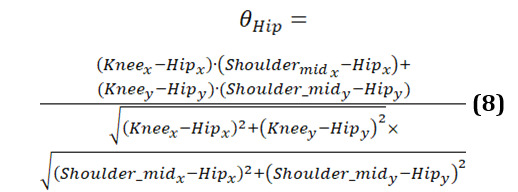



The graphs showing the cosine of the hip angle are shown in Figure 12. The figure shows 3-line graphs comparing hip angles during body turns for the 3 groups. Each graph represents the hip angles for both the left and right sides, with lines depicting both the original and smoothed data.

The graph of the younger individuals group (Figure 12A) indicates relatively stable and moderate fluctuations in hip angles during body turns. Both hips show consistent movement with shallow peaks and troughs, reflecting balanced and controlled motion. Younger individuals maintain a steady range of hip angles, suggesting efficient and coordinated hip movement when turning.

In the older-individual group graph (Figure 12B), the hip angles display more pronounced and variable fluctuations compared to the young group. The curves reveal that older individuals experience larger and more variable hip movements during turns. This increased variability suggests that older people adjust their hip movements significantly to maintain balance and stability, resulting in less smooth and more fluctuating hip angles.

The group of individuals with PD (Figure 12C) shows significant irregularity and instability in hip angles. The curves exhibit erratic and sharp changes, highlighting the difficulty individuals with PD face in maintaining consistent hip movement during turns. The frequent and abrupt fluctuations in hip angles are characteristic of the disease, where symptoms such as rigidity and tremors disrupt smooth and coordinated movements.

**Figure 13:**
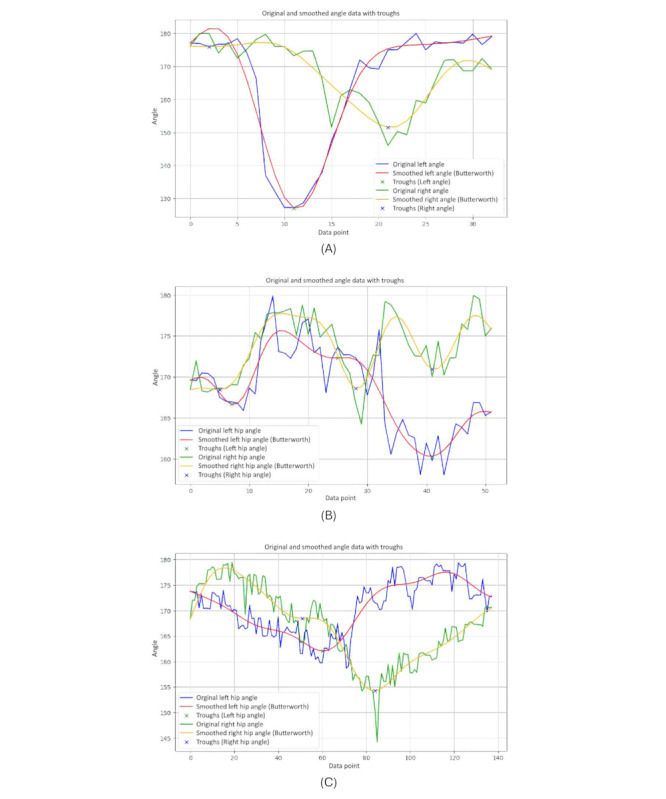
A comparative analysis of the hip angle between different groups: (A) younger individuals, (B) older individuals, and (C) individuals with Parkinson disease.

Figure 13 illustrates clear differences in hip angle dynamics during turns among the 3 groups. Younger individuals (Figure 13A) have controlled and consistent hip movements, older individuals (Figure 13B) show more variability and larger fluctuations in hip angles, and those with PD (Figure 13C) experience erratic and unstable hip movements. These patterns reflect how age and neurological conditions affect the coordination and efficiency of hip movement during body turns.

### Leg Symmetry

**Figure 14:**
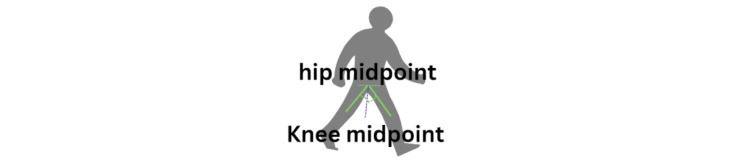
An illustration of the symmetrical leg position.

The symmetry of leg movements is calculated using the angles at the knee joints (Figure 14). The goal was to measure and evaluate the balance and symmetry in the leg movements, which is crucial for understanding gait and stability, especially during turning motions. This information is particularly useful for analyzing the movement patterns in different populations, such as detecting gait irregularities in individuals with conditions such as PD.

The leg angle is computed as follows (equations 9-11):



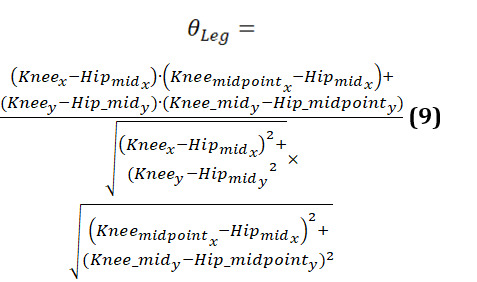



Next, the angles of the left and right legs are compared:



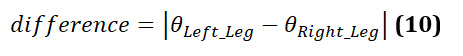



The leg symmetry is then assessed as:



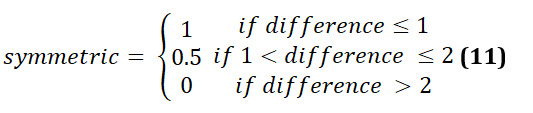



**Figure 15:**
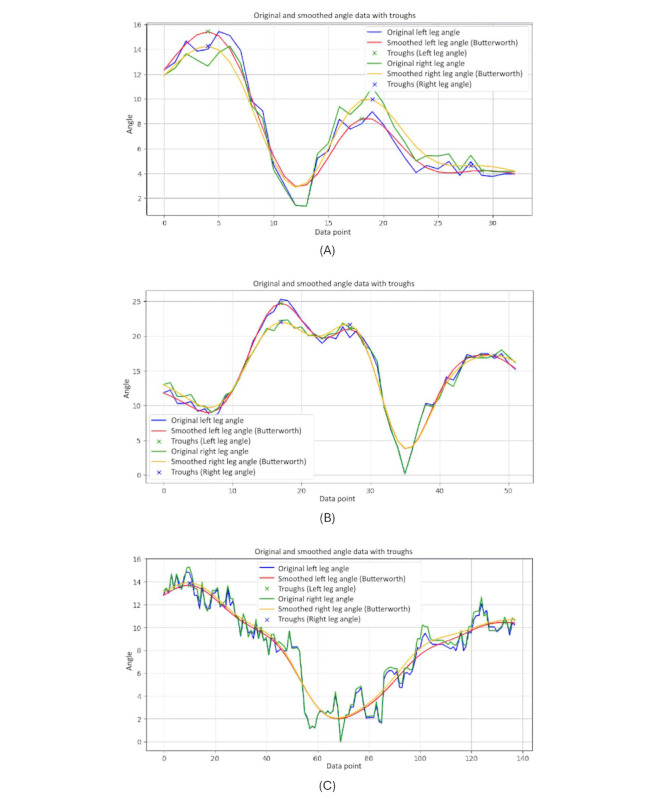
A comparative analysis of the symmetrical leg between different groups: (A) younger individuals, (B) older individuals, and (C) individuals with Parkinson disease.

Figure 15 displays 3-line graphs comparing the leg symmetry during body turns for the 3 participant groups. Each graph plots the symmetrical leg movements, showing the original and smoothed angles of the left and right legs as they turn.

The graph of the younger-individual group (Figure 15A) shows closely aligned curves for the left and right leg angles, indicating high symmetry in their leg movements during turns. Both legs exhibit similar patterns with minimal deviation between them. This close alignment reflects the balanced and coordinated movements typical of young, healthy individuals, suggesting that they maintain a consistent and symmetrical gait.

The older-individual group graph (Figure 15B) reveals more noticeable fluctuations between the left and right leg angles. While the overall patterns still follow a similar trajectory.

On the other hand, the graph of individuals with PD (Figure 15C) shows significant irregularities and less alignment between the left and right leg angles. The curves are erratic and display frequent sharp deviations, reflecting low symmetry in their leg movements. This lack of alignment is characteristic of PD, where symptoms such as tremors and rigidity cause disrupted and uncoordinated movements.

This comparison highlights how age, neurological conditions, and health status impact the ability to maintain balanced and symmetrical gait during turning motions.

### Arm Symmetry

**Figure 16:**
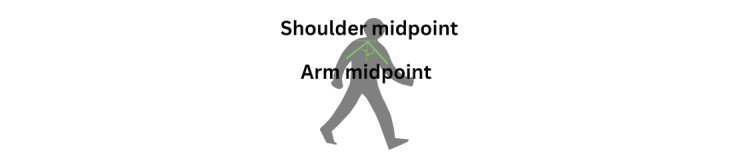
An illustration of the symmetrical arm position.

The symmetry of arm movements was analyzed using the angles at the elbows to the shoulders (Figure 16). The purpose was to measure how balanced and coordinated the arm movements are, especially during activities such as turning the body. This assessment was important for understanding the ROM and the symmetry in arm movements, which can provide insights into overall body coordination and detect possible imbalances or movement disorders.

The calculation for arm symmetry was similar to that of finding the symmetry in legs (equation 12):



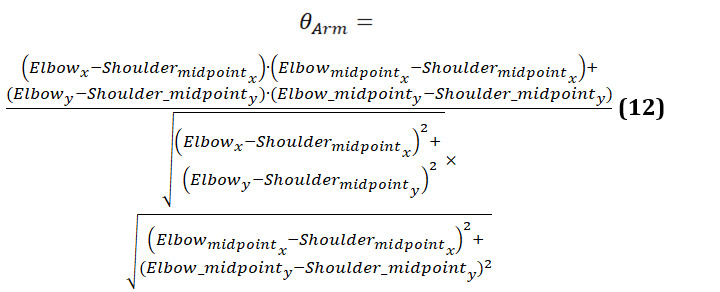



**Figure 17:**
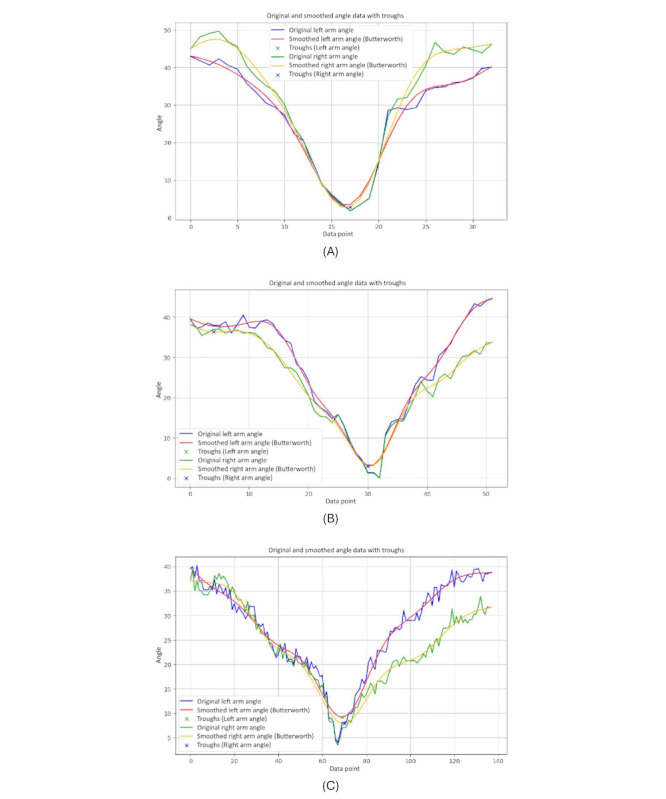
A comparative analysis of the symmetrical arms between different groups: (A) younger individuals, (B) older individuals, and (C) individuals with Parkinson disease.

Figure 17 presents 3-line graphs comparing the arm symmetry during body movements for 3 groups. Each graph plots the angles of the left and right arms over time, showing both the original and smoothed data.

The graph of the younger-individual group (Figure 17A) displays closely aligned curves for the left and right arm angles. Both arms move in a highly synchronized manner, with minimal differences between the left and right angles throughout the motion.

The older-individual group graph (Figure 17B) shows a slightly more varied pattern compared with the young group. While the overall movements of the left and right arms remain relatively synchronized, there are noticeable differences in the magnitude and timing of the angles.

The graph of individuals with PD (Figure 17C) exhibits significant irregularities and discrepancies between the left and right arm angles. The curves are erratic and often diverge sharply, indicating substantial asymmetry in arm movements.

We observed that younger individuals demonstrate highly synchronized and symmetrical arm movements, older individuals show moderate symmetry with some variability, and those with PD experience significant asymmetry and irregularity in their arm movements. These patterns underscore how age and neurological conditions impact the coordination and balance of arm movements during body turns.

### Trunk Angle 1 (Vertical)

**Figure 18:**
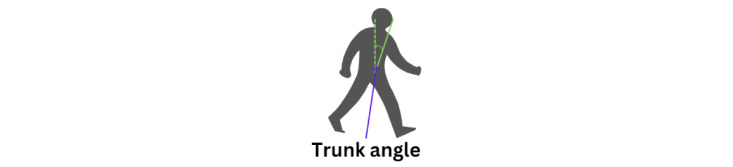
An illustration of the trunk angle 1 vertical position.

The relative positions of the hips and nose are used to calculate the trunk angle (Figure 18). The trunk angle helps to understand how much the upper body tilts or bends relative to the lower body, especially during activities such as turning. This angle helps in assessing posture and balance, providing insights into the coordination and alignment of the trunk with the hips during movement. It is particularly useful in evaluating movement patterns and detecting postural deviations in various populations, including those with movement disorders such as PD.

The calculation of the trunk angle 1 is given as follows (equation 13):



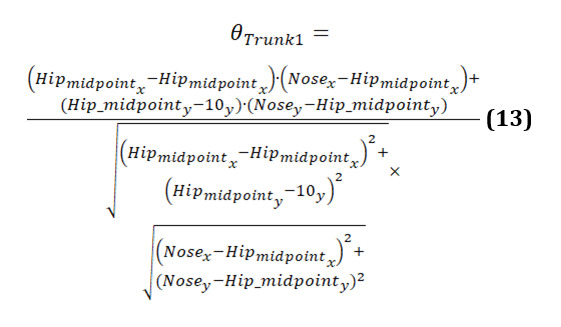



**Figure 19:**
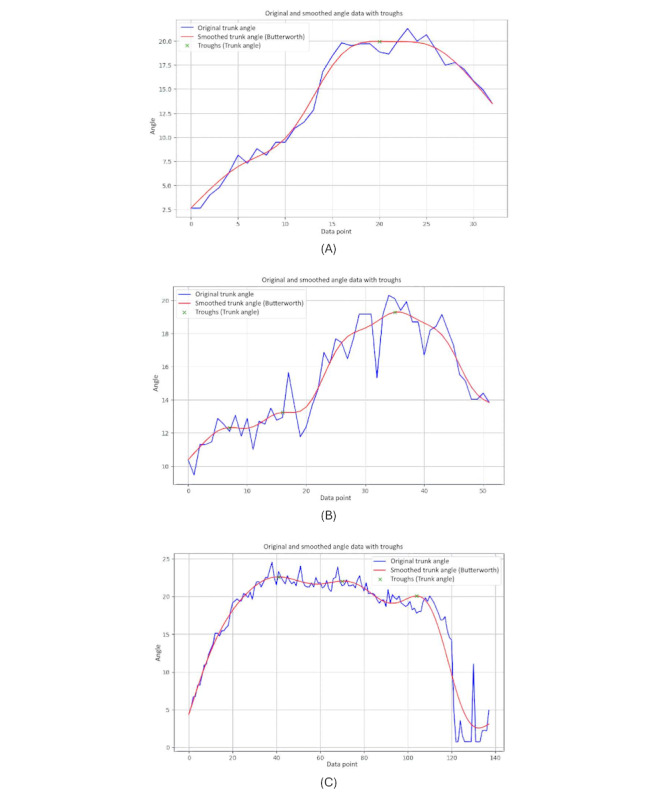
A comparative analysis of the trunk angle 1 between different groups: (A) younger individuals, (B) older individuals, and (C) individuals with Parkinson disease.

Figure 19 displays 3 graphs comparing trunk angles relative to the vertical axis for 3 groups. Each graph shows the trunk angle over a series of data points during body movements, with both original and smoothed data represented. The trunk angle is used to observe the severity of hunchback, providing insights into the degree of forward bending or curvature of the upper body.

The graph of the younger-individual group (Figure 19A) exhibits a consistent and smooth progression of trunk angles. The trunk angle gradually increases and decreases within a narrow range, reflecting a well-coordinated and balanced posture with minimal forward bending. Younger individuals maintain a steady alignment with minimal deviations, indicating a lack of significant hunchback severity.

The older-individual group graph (Figure 19B) shows more variability in trunk angles compared to the young group. The curves display noticeable fluctuations and less consistency, with several abrupt changes in angle. These variations suggest that older individuals experience more difficulty in maintaining a stable trunk posture, leading to less smooth and more erratic movements. The increased trunk angles and variability may indicate a greater tendency toward forward bending, suggesting a moderate severity of hunchback as they struggle to maintain an upright posture.

The graph of individuals with PD (Figure 19C) reveals significant irregularities and instability in trunk angles. The curves are highly erratic, with frequent sharp changes and a wide range of deviations from the vertical alignment. These abrupt shifts indicate substantial difficulties in maintaining consistent trunk posture. Individuals with PD struggle with controlling their trunk movements, leading to frequent tilting and misalignment relative to the vertical axis. The pronounced forward bending and high variability in trunk angles reflect severe hunchback, characteristic of the disease’s impact on posture and movement control.

### Trunk Angle 2 (Horizontal)

**Figure 20:**
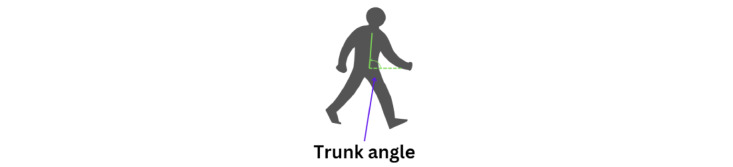
An illustration of the trunk angle 2 horizontal position.

Trunk angle 2 analyzes the positions of the shoulders and hips (Figure 20). This angle is crucial for understanding the alignment of the trunk relative to the lower body, particularly during movements such as turning. By evaluating the trunk angle, we can assess the severity of hunchback or forward bending, which provides valuable insights into posture and stability.

The calculation for trunk angle 2 is similar to that for trunk angle 1. The only difference is that as trunk angle 2 is horizontal, the default value must be set at the *x* coordinate instead of the keypoint. The formula to calculate trunk angle 2 is given as follows (equation 14):



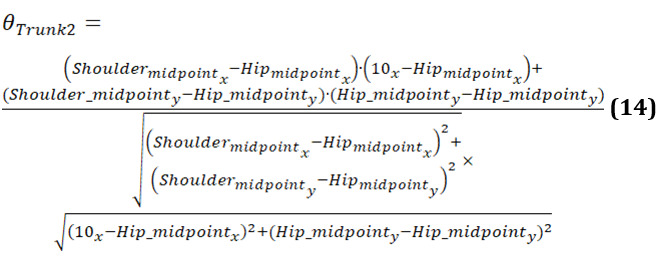



**Figure 21:**
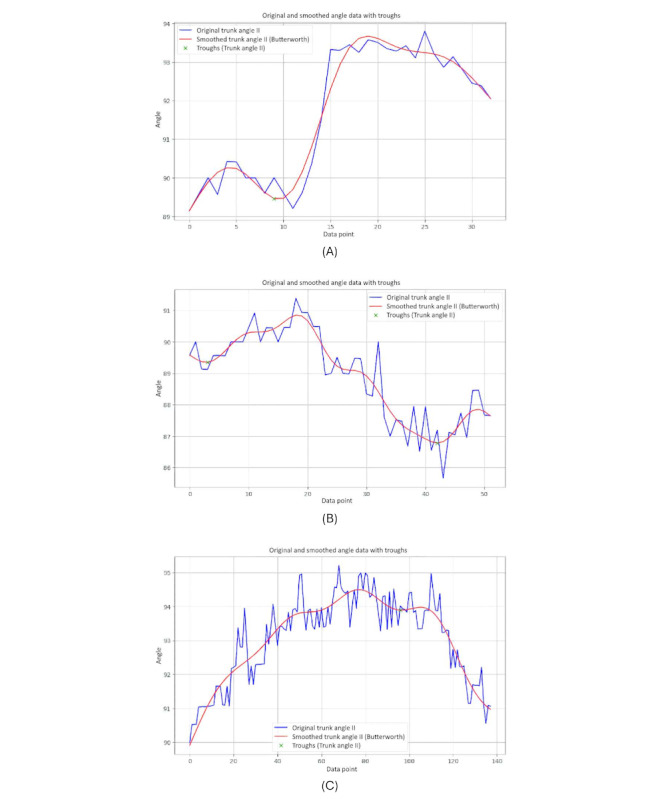
A comparative analysis of the trunk angle 2 horizontal between different groups: (A) younger individuals, (B) older individuals, and (C) individuals with Parkinson disease.

Figure 21 shows graphs comparing trunk angles relative to the horizontal axis for 3 groups. These angles help assess the severity of forward bending or misalignment.

The graph of the younger-individual group (Figure 21A) exhibits stable and controlled trunk angles with minimal deviations, indicating well-balanced posture and low severity of hunchback.

The graph of the older individuals’ group (Figure 21B) showed more variability and sudden changes in trunk angle, reflecting moderate forward lean and greater difficulty maintaining stable horizontal alignment, indicating moderate kyphosis severity.

The graph of the individuals with PD (Figure 21C) shows significant instability and frequent sharp changes in trunk angles, indicating severe difficulties in maintaining consistent trunk posture and severe hunchback severity due to the disease’s impact on movement control.

Overall, younger individuals maintain a low severity of hunchback, older individuals exhibit moderate severity, and those with PD show severe hunchback, reflecting their challenges in maintaining horizontal alignment.

### Shank Angle

**Figure 22:**
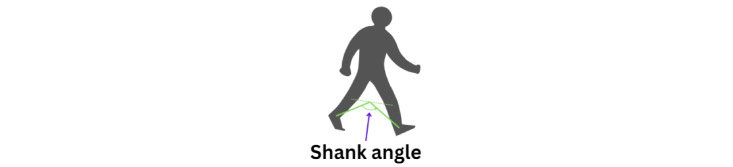
An illustration of the shank angle position.

The shank angle computes the positions of the knees and ankles (Figure 22). This angle is useful to understand the alignment and movement of the lower leg, especially during activities such as turning. By evaluating the shank angle, we can assess the coordination and stability of the lower leg movements, providing valuable insights into gait patterns and detecting any abnormalities in movement, which is particularly useful for analyzing conditions such as PD.

The formula to calculate shank angle is provided in equation 15:



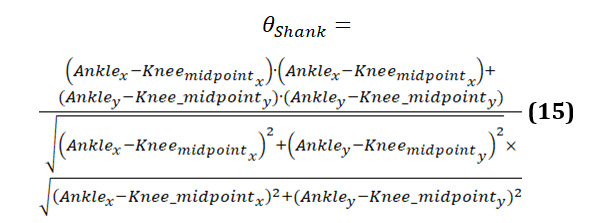



**Figure 23:**
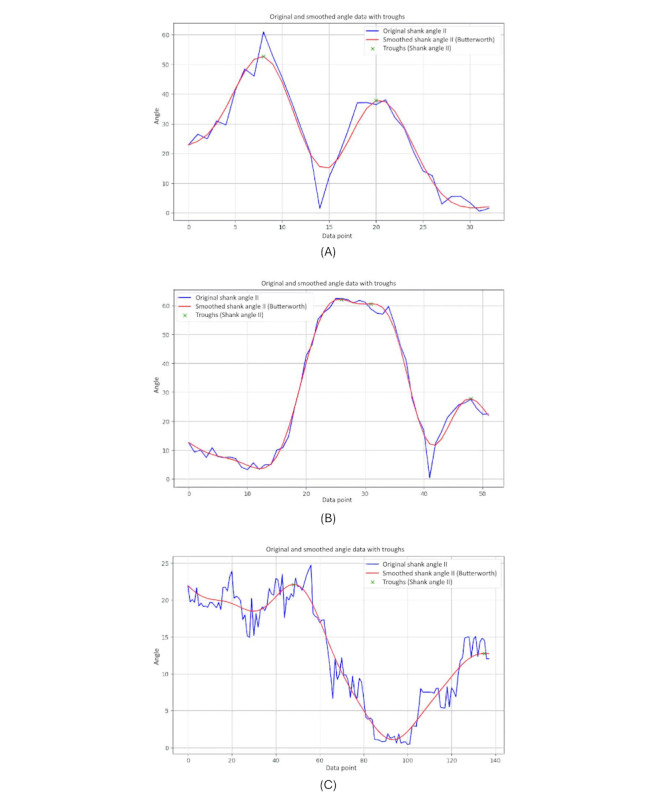
A comparative analysis of the shank angle between different groups: (A) younger individuals, (B) older individuals, and (C) individuals with Parkinson disease.

Figure 23 presents the graphs comparing shank angles during body movements for 3 groups. Each graph shows the shank angle over a series of data points, with both original and smoothed data represented.

The graph of the younger-individual group (Figure 23A) demonstrates relatively stable and consistent shank angles. Both the original and smoothed curves align closely, indicating smooth and coordinated movements. The angles show moderate fluctuations within a narrow range, reflecting balanced and controlled lower leg movements typical of healthy young individuals.

The older-individual group graph (Figure 23B) shows shank angles that are stable and consistent, such as the younger individuals group. Both the original and smoothed curves align closely, indicating smooth and coordinated lower leg movements. The angles show moderate fluctuations within a narrow range, reflecting balanced and controlled movements. This suggests that older individuals perform nicely, maintaining stable shank angles comparable to the younger individuals group.

The graph of individuals with PD (Figure 23C) reveals significant irregularities and instability in shank angles. The curves are highly erratic, with frequent sharp changes and a wide range of deviations. These abrupt shifts indicate substantial difficulties in maintaining consistent lower leg movements. Individuals with PD struggle with controlling their shank angles, leading to frequent misalignment and instability. This pattern is characteristic of the disease, where symptoms such as rigidity and tremors disrupt smooth and coordinated movements.

Figure 23 highlights the differences in shank angle behavior among the 3 groups. Younger and older individuals exhibit stable and coordinated shank movements with minimal variability, indicating balanced and controlled lower leg movements. In contrast, those with PD display significant instability and erratic shank movements, underscoring the impact of the condition on lower leg control and stability.

### Classification Report

A comprehensive evaluation of the SVM model is provided by the classification report for the test set, as shown in Table 3. The report includes the overall accuracy of the model, as well as each class’s recall, precision, and *F_1_*-score. Although the training accuracy was 0.95, the test accuracy was 0.89, indicating that the model fit the training data well with minimal overfitting. The classification report shows that class 0 (normal class) had high precision and recall, while class 1 (PD class) has lower recall. This disparity in recall may be due to the smaller sample size of class 1.

**Table 3:** Classification report for distinguishing normal and PD^a^ classes using gait features (test accuracy: 0.89 and train accuracy: 0.95).

Classes or metrics	Precision	Recall	*F*_1_-score	Support
0 (normal)	0.88	1.00	0.93	7.00
1 (PD)	1.00	0.50	0.67	2.00
Accuracy	—^b^	—	0.89	9.00
Macroaverage	0.94	0.75	0.80	9.00
Weighted average	0.90	0.89	0.87	9.00

^a^PD: Parkinson disease.

^b^Not available.

### AUC and Loss Curve

**Figure 24:**
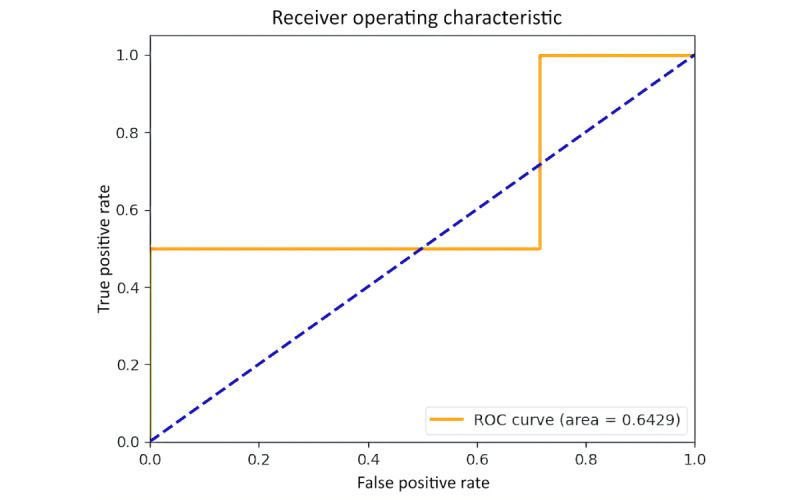
The receiver operating characteristic (ROC) curve.

Figure 24 provides specifics regarding the model’s capacity to distinguish between the 2 classes. Moderate discrimination capability is indicated by the test set’s AUC of 0.6429. An indicator of how well the model performs across various training data subsets is provided by the cross-validation scores. With a mean cross-validation score of 0.9, the model’s cross-validation scores are (0.75, 1.00, 1.00, 0.75, 1.00). This shows that the model continues to work well when the data are folded in different ways.

**Figure 25:**
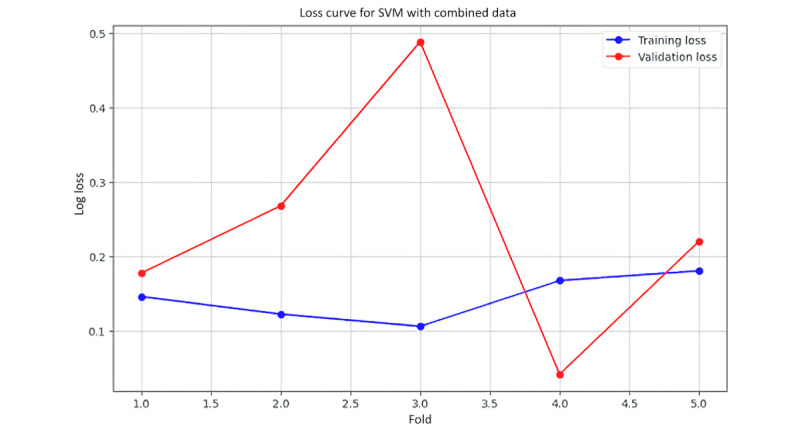
The training and validation loss curve for support vector machine (SVM) with combined data.

Figure 25 shows the training and validation loss curve, illustrating the log loss for both training and validation sets across various folds of cross-validation. The blue curve indicates the training loss, which stays relatively stable with minor fluctuations. In contrast, the red curve, representing the validation loss, exhibits significant fluctuations, peaking notably at fold 3. This implies that while the model fits the training data well, it experiences considerable variability in performance on unseen data, suggesting possible overfitting or sensitivity to specific data subsets. The difference between the training and validation loss also underscores the model’s difficulty in generalizing from the training set to the validation set, highlighting the need for potential adjustments in model complexity or training strategy to achieve more consistent performance.

### Confusion Matrix

**Figure 26:**
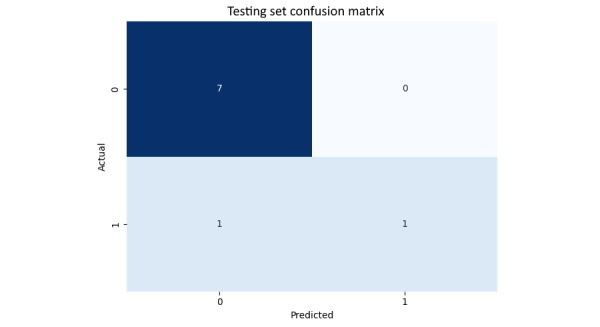
The testing set confusion matrix for model-based features.

The confusion matrix depicted in Figure 26 provides a thorough analysis of the predictions of the model on the test set. It reveals that while the model correctly predicted 7 out of 7 occurrences of class 0 (non-PD), it incorrectly classified 1 out of 2 instances of class 1 (PD). This suggests that although the model is highly accurate in predicting class 0, it faces challenges with class 1, likely due to class imbalance in the dataset.

## Discussion

This study presents a noninvasive approach for early detection of PD through the analysis of model-based gait features. Using kinematic characteristics, such as shoulder distance, step length, stride length, knee and hip angles, leg and arm symmetry, and trunk angles, we aimed to identify subtle gait abnormalities associated with PD. Data were collected through controlled video recordings of the TUG assessment, and the extracted features were processed using advanced filtering techniques and analyzed using SVM classifier.

The results demonstrate that the model-based features were highly effective in distinguishing between normal and PD-affected gait patterns, achieving high accuracy, precision, recall, and *F*_1_-score. These findings support the potential of model-based gait analysis as a noninvasive, accessible tool for early diagnosis and monitoring of PD. The study also highlights the importance of addressing class imbalance and refining the feature extraction process to further enhance the performance of the classifier.

Future research should focus on validating these findings with larger datasets, exploring other machine learning models, and integrating additional features to improve the robustness and accuracy of PD detection systems. This approach could significantly benefit clinical settings, providing a reliable, noninvasive method for early diagnosis and improved patient outcomes.

## Abbreviations

ANN: artificial neural network
AUC: area under the curve
CNN: convolutional neural network
DNN: deep neural network
FoG: freezing of gait
KNN: k-nearest neighbors
LSTM: long short-term memory
PD: Parkinson disease
ROM: range of motion
SVM: support vector machine
TUG: timed up and go
UPDRS: Unified Parkinson Disease Rating Scale

## References

[1] Challa KN, Pagolu VS, Panda G, Majhi B (2016). An improved approach for prediction of Parkinson's disease using machine learning techniques. Proceedings of the International Conference on Signal Processing, Communication, Power and Embedded System.

[2] Polat K (2019). Freezing of gait (FoG) detection using logistic regression in Parkinson's disease from acceleration signals. Proceedings of the Scientific Meeting on Electrical-Electronics & Biomedical Engineering and Computer Science.

[3] Vidya B, Sasikumar P (2021). Gait based Parkinson’s disease diagnosis and severity rating using multi-class support vector machine. Appl Soft Comput.

[4] Fang Z (2022). Improved KNN algorithm with information entropy for the diagnosis of Parkinson's disease. Proceedings of the International Conference on Machine Learning and Knowledge Engineering.

[5] Gundala S, Harichandana C, Jaasmitha A, Brundha PL (2023). Parkinson’s disease identification by voice and handwritten drawings using Xgboost and Random Forest Algorithms. Proceedings of the 3rd International Conference on Computing and Information Technology.

[6] Govindu A, Palwe S (2023). Early detection of Parkinson's disease using machine learning. Procedia Comput Sci.

[7] Pereira CR, Weber SA, Hook C, Rosa GH, Papa JP (2016). Deep learning-aided Parkinson's disease diagnosis from handwritten dynamics. Proceedings of the 29th SIBGRAPI Conference on Graphics, Patterns and Images.

[8] Grover S, Bhartia S, Yadav A, KR S, Akshama (2018). Predicting severity of Parkinson’s disease using deep learning. Procedia Comput Sci.

[9] Aal HA, Taie S, El-Bendary N (2021). An optimized RNN-LSTM approach for Parkinson’s disease early detection using speech features. Bull Electr Eng Inform.

[10] Ouhmida A, Terrada O, Raihani A, Cherradi B, Hamida S (2021). Voice-based deep learning medical diagnosis system for parkinson's disease prediction. Proceedings of the International Congress of Advanced Technology and Engineering.

[11] Biswas S, Kaur N, KR S (2022). Early detection of Parkinson’s disease from hand drawings using CNN and LSTM. Proceedings of the 4th International Conference on Artificial Intelligence and Speech Technology.

[12] Khaskhoussy R, Ayed YB (2023). Improving Parkinson’s disease recognition through voice analysis using deep learning. Pattern Recognit Lett.

